# Trends in waking salivary alpha-amylase levels following healing lucid dreams

**DOI:** 10.3389/fpsyg.2024.1347499

**Published:** 2024-01-17

**Authors:** Garret Yount, Sitara Taddeo, Tadas Stumbrys, Michael Kriegsman, Helané Wahbeh

**Affiliations:** ^1^Institute of Noetic Sciences, Novato, CA, United States; ^2^Institute of Psychology, Vilnius University, Vilnius, Lithuania

**Keywords:** PTSD, salivary biomarker, dreaming, lucidity, trauma, nightmares

## Abstract

**Introduction:**

Salivary alpha-amylase (sAA) is considered a marker of autonomic nervous system activity in stress research, and atypical waking sAA responses have been reported for traumatized individuals. Lucid dreams, characterized by a dreamer’s awareness of their dream state while remaining asleep, have shown promising preliminary evidence of their potential to enhance mental health. This study’s objective was to evaluate sAA in relation to healing lucid dreams.

**Methods:**

Participants experiencing PTSD symptoms attended a six-day workshop delivered via live video designed to teach techniques for transforming trauma through dreamwork and dream lucidity. Participants (*n* = 20) collected saliva samples each morning, immediately upon awakening (Time 1) and 30 min afterward (Time 2). sAA levels were determined by enzymatic assay, and the waking sAA slope was calculated as the difference of Time 2 minus Time 1. Participants completed dream reports each morning, with a dream classified as a ‘healing lucid dream’ when they reported attaining lucidity and remembered their intention to manifest a healing experience within the dreamscape.

**Results:**

Of eight participants experiencing healing lucid dreams, four were able to provide usable saliva samples. Statistical tests on these four participants were not significant because of low power. However, nonsignificant positive associations were observed between experiencing more healing lucid dreams and increased waking sAA slope.

**Conclusion:**

The results did not reveal a consistent effect of healing lucid dreams on waking sAA slope. Identifying meaningful patterns in this relationship will require larger samples and more stringent control over saliva collection procedures in future studies.

## Introduction

As of 2023, current estimates forecast one in eleven Americans will struggle with post-traumatic stress disorder (PTSD) at some point in their lives (Taylor-Desir, 2022). Mild symptoms include altering the ability to concentrate, impairing memory, disrupting sleep, and increasing irritability or startle response. More severe symptoms can manifest as social isolation, looping or racing negative thoughts, flashbacks, increased propensity of risk-taking behavior, and difficulty experiencing positive emotions (Taylor-Desir, 2022). The severe nervous system dysregulation of PTSD is often intrusive and disruptive to daily life for those with this diagnosis. Furthermore, relief from pharmaceuticals often falls short for this complex condition (Krystal et al., 2017). A novel avenue for therapeutic relief may be found in the practice of lucid dreaming, wherein the dream state opens a conduit between the subconscious and conscious mind, granting access to previously untapped realms of the psyche (Jung, 1968; Priya and Jain, 2021).

The belief in the healing power of dreams has existed in various ancient cultures throughout history and is still prevalent today. Ancient Grecians seeking cures for ailments would sleep within temples dedicated to Asclepius, the god of healing, expecting to receive healing dreams (Rousselle, 1985). Similarly, ancient Egyptian medical texts reference the healing power of dreams (Asaad, 2015). Carl Jung wrote about the concept of “healing dreams” as an integral part of his work on dream analysis and psychology. He believed dreams to be a window into the unconscious mind and a means of integrating conscious and unconscious aspects of an individual’s psyche (Jung, 1968). Currently, some practitioners of Ayurveda, the traditional system of medicine in India, use dream analysis as a diagnostic tool and may recommend treatments or changes in behavior based on dream content (Munishwar et al., 2019). Native American Nations also incorporate dream quests and interpretation in their healing practices (Georgiou, 2002). These few examples illustrate the widespread belief in the healing potential of dreams in both ancient and modern cultures. Lucid dreaming has the potential to increase the effectiveness of healing dreams.

Being lucid in a dream refers to being consciously aware while within a dream and can allow the dreamer to influence what happens in the dream. Though the concept of realizing one’s dream state during sleep has ancient roots (LaBerge, 1988; Wallace and Hodel, 2012), the term lucid dream (LD) was first introduced by the Dutch psychiatrist Frederik van Eeden in 1913 (Van Eeden, 1913). Since then, numerous studies have documented the experience and its neurophysiological concomitants (Baird et al., 2019).

Most important for this study is the therapeutic potential of lucid dreaming for enhancing mental health. The idea that a dreamer’s self-reflective awareness within a LD can be directed toward self-development is a central tenet of the Tibetan practice of dream yoga (Wangyal, 1998). Perhaps as an extension of this tenet, about 40% of modern lucid dreamers report using LDs to improve their physical and mental health (Stumbrys and Erlacher, 2016). Further, most lucid dreamers believe that their LDs contribute to their mental and physical health (Erlacher et al., 2020). The possible mental health benefits of lucid dreaming have also been suggested by a retrospective study highlighting the potential of lucid dreaming treatment for clinical depression (Sackwild and Stumbrys, 2021), as well as a pilot study showing that lucid dreaming can contribute to managing insomnia by reducing its severity as well as anxious and depressive symptomatology (Ellis et al., 2021). In relation to PTSD, the effects of lucid dreaming treatment, while promising, are somewhat inconclusive (Spoormaker and van den Bout, 2006; Holzinger et al., 2020). However, a recent pilot study evaluating the efficacy of a healing lucid dream (HLD) workshop found that the participants achieved sustained relief from symptoms of PTSD compared to before the workshop (Yount et al., 2023). The HLD pilot study also presented preliminary data supporting the feasibility of quantifying levels of a physiological biomarker using saliva samples collected from participants before and after the workshop.

Identifying reliable physiological biomarkers associated with HLDs would be a significant step forward in understanding the mechanism of action underlying their therapeutic potential for mental health. Such biomarkers could help validate and refine the applicability of HLDs as a clinical intervention for conditions such as PTSD and depression, ultimately improving the well-being of those who suffer from these disorders. A promising biomarker in this regard is salivary alpha-amylase (sAA) because alterations in the waking sAA slope have been associated with stress and PTSD (Nater and Rohleder, 2009; Thoma et al., 2012; Nicholson et al., 2014). While the first identified physiological function of sAA was the initiation of the breakdown of starch in the oral cavity, levels of this enzyme in the saliva have proven useful as a stress biomarker due to its association with increased catecholamine activity of the sympathetic nervous system (Nater et al., 2007; Schumacher et al., 2022). The pilot study evaluating the efficacy of a HLD workshop found that the waking sAA slopes for two participants on the morning following a HLD were decreased compared to two workshop participants who did not have a HLD (Yount et al., 2023).

Here, we report on an exploratory analysis of waking sAA slopes in a subgroup of participants from a larger randomized controlled study evaluating PTSD symptoms before and after a HLD workshop (Yount et al., 2024). The rationale behind our randomized controlled study and the plan for this exploratory analysis of waking sAA slopes was informed by preliminary results from the HLD pilot study, indicating a sustained alleviation of PTSD symptoms for up to 3 weeks following the HLD workshop (Yount et al., 2023). Notably, most participants in the HLD pilot study experienced only one HLD throughout the data collection period. This observation sparks the intriguing possibility that a single HLD could potentially confer enduring therapeutic benefits, leading to lasting alterations in associated physiological biomarkers. Consequently, our first hypothesis sought to investigate whether a single HLD could indeed induce detectable changes in waking sAA slope. Our focus was on examining the impact of the first HLD experience on waking sAA slopes and the day-to-day changes in this slope, as they might be potential points of effect. Considering the possibility that no such impact is detected, we reasoned that this might be attributed to the actual lack of such an effect, or methodological factors introducing noise to the outcome measure. Hence, our second hypothesis aimed to broaden the inquiry by investigating whether increasing numbers of HLD could lead to detectable changes in waking sAA slope.

Taking the inquiry a step further, our third hypothesis delved into quantifying the intensity of HLDs. Although no precedent exists for aggregating HLD intensities, the decision was influenced by observations in the HLD pilot study, where participants reported varied subjective impacts from their dream experiences (Yount et al., 2023). Our interest was in discerning whether these subjective variations in intensity could be associated with differing effects on waking sAA slope. Since these analyses are in somewhat uncharted territory, we adopted an exploratory approach for our fourth hypothesis, involving the generation of line plots to visually capture day-to-day variations in sAA levels. This strategy aimed to reveal potential patterns in the data that might not be evident through more traditional analytical methods.

## Method

### Participants

Participants for the analyses reported here were recruited as part of a larger randomized controlled study (please see Yount et al., 2024, for full study design details). In brief, eligible participants were at least 18 years of age and experiencing PTSD symptoms as determined through self-report, with PCL-5 scores (see Measures below) ranging from 28 to 59. Anyone experiencing self-reported PTSD was permitted to participate in the study, resulting in a heterogeneous population in the origin of their PTSD (see Supplementary materials). Exclusion criteria included pregnancy, regular use of sleeping pills, and past or present psychotic episodes (e.g., visual or auditory hallucinations). To avoid disrupting any ongoing therapeutic regimens, medications or supplements participants were currently taking aside from sleeping pills and antipsychotics were not considered exclusion criteria. All participants in the randomized controlled study were given a Muse EEG device to use and keep in exchange for taking part in the study ($400 value). The study design was preregistered (osf.io/ne78g), and all activities were approved and overseen by the Institutional Review Board (IRB) at the Institute of Noetic Sciences (IORG#0003743).

A subset of participants (*n* = 26) enrolled in the parent study (*N* = 100, Yount et al., 2024) were invited to collect saliva samples based on their location during the workshop. Eligible participants for this subgroup were limited to the continental United States to enable cost-effective transport and controlled temperatures while in transit. Of those invited, 20 accepted and collected saliva samples. Participants were instructed not to brush their teeth, smoke, eat, or drink anything other than water before sample collection to maintain sample viability. Samples were kept in a household freezer throughout the workshop. Then, samples were picked up from participants’ homes and transported to the laboratory on dry ice by a medical courier service. Six participants’ samples were compromised due to mishandling, and one participant dropped out, leaving samples from 13 participants available for analysis. All participants collecting saliva samples were also given a book ($15 value).

### Intervention: healing lucid dream (HLD) workshop

The intervention consisted of an intensive online HLD workshop designed to teach lucid dreaming induction techniques with didactic instruction and group activities, conducted via Zoom video conferencing and spread over a six-day period (five and a half hours per day on the first 2 days and 2 h per day for the remainder of the workshop). The instructor has over 20 years of experience teaching lucid dreaming and is “authorized to teach” within the Kagyu School of Tibetan Buddhism. The instruction included neuroscience principles of sleep and dreaming, mindfulness practices for deep relaxation, sleep hygiene principles, practices to increase dream recall, and multiple lucid dreaming induction techniques presented in sequence so that the participants could attempt a novel technique each night. The induction techniques included attention to dream signs, reality checks, wake-up-back-to-bed sleep protocol, mnemonic technique, and falling asleep consciously (Stumbrys et al., 2012). Some participants set an alarm to wake up during the night, after which they tried to recall their dreams and were encouraged to listen to audio recordings designed to reinforce induction techniques as they fell back asleep. Activities also integral to the workshop (both during the live sessions and on their own in between sessions) included guided meditations, yoga Nidra sessions, dream-sharing circles, LD exercises, and dream planning lessons. Data were collected from two participant cohorts, each attending identical HLD workshops taught by the same instructor (one in January 2023 and one in April 2023). A psychotherapist accredited in Mindfulness-based Core Process Psychotherapy through the United Kingdom Council for Psychotherapy was present during the live instruction and available throughout the entire workshop period for private participant consultations.

### Measures

#### Life events checklist for DSM-5 (LEC)

The LEC assesses exposure to 16 events known to result in PTSD or potential distress and includes one additional item assessing any other extraordinarily stressful event not captured in the first 16 items (Weathers et al., 2013). Respondents check one or more boxes for each event to indicate whether the event (a) happened to them personally; (b) they witnessed it happen to someone else; (c) they learned about it happening to a close family member or close friend; (d) they were exposed to it as part of their job (e.g., paramedic, police, military, or other first responder); (e) they are not sure if it fits; or (f) it does not apply to them.

#### PTSD checklist for DSM-5 (PCL-5)

The PCL-5 is a 20-item measure that assesses the 20 symptoms of PTSD listed in the Diagnostic and Statistical Manual of Mental Disorders, Fifth Edition (Blevins et al., 2015).

#### Drug questionnaire

A simple questionnaire was given to participants, asking them to please indicate any medications or supplements they were currently taking that may impact their sleep or dream experiences during the study.

#### Dream report

Participants recorded any LDs experienced the previous night through a survey completed each morning using their personal devices. The workshop included explicit definitions of lucid dreaming to ensure a clear understanding of the concept (see Supplementary material for example).

#### Dream lucidity questionnaire (DLQ)

After reporting their LDs, participants recorded the intensity of each dream using the DLQ (Stumbrys et al., 2013). Each LD was rated by 12 items ranging from 0 (“not at all”) to 4 (“very much”) to indicate the degree to which they experienced that characteristic during the dream. The DLQ evaluates different types of awareness (such as awareness of the dream state, the awareness of the physical body’s dormancy, recognition that dream elements are not real, and awareness of multiple possibilities) and control within the dream (involving the deliberate selection of actions, alteration of dream events, manipulation of dream characters and settings, and the transgression of physical laws). A score of ‘HLD intensity’ was computed for each HLD as the average of 10 of the 12 DLQ questions. Questions #7 and #12 were excluded from scoring because of a previous report that they loaded poorly in factor analysis (i.e., <0.4), and following precedent in the literature (Gott et al., 2021).

#### Criterion for a healing lucid dream (HLD)

Question #12 of the DLQ was altered to assess the participants’ recollection of the intention to heal within the lucid dream; it read: “I clearly remembered my intention that I wanted to do in a lucid dream (i.e., healing).” A non-zero response to this question was used as the criterion to differentiate a LD from a HLD, following precedent in the literature (Yount et al., 2023). Thus, we used the DLQ to categorize dreams into three types: (1) non-lucid dream, (2) non-healing LD, and (3) HLD.

#### Salivary alpha-amylase (sAA)

Saliva samples were collected using oral swabs (SalivaBio Oral Swab Device; Salimetrics, State College, PA) at awakening (Time 1) and 30 min later (Time 2) each morning of the workshop and the morning following the last workshop day (14 samples per participant). Samples were immediately placed into their household freezer for storage until the end of the workshop. The samples were then transferred to the Salimetrics laboratory on dry ice via a medical courier service. sAA levels were assayed using a kinetic reaction method with a sensitivity of 0.4 U/mL and a range of 2–400 U/mL in duplicate. On each morning, the ‘waking sAA slope’ was computed by subtracting the value of Time 2 from Time 1. The ‘day-to-day change in waking sAA slope’ was calculated by subtracting the waking sAA slope of a given day from the waking sAA slope value of the previous day. The ‘pre-post change in waking sAA slope’ was computed by subtracting the waking sAA slope value of Day 7 minus Day 1, in order to capture the overall effect of the workshop.

#### Timing of measures

The LEC and drug questionnaire were administered one time before the start of the workshop. The PCL-5 was administered before and immediately after the workshop and again 3 weeks after the workshop’s conclusion. The dream reports (including the DLQ) were collected every morning during the workshop and the morning after the workshop ended (Day 7).

### Statistical analysis

Preregistered analyses were organized into four hypotheses. Hypothesis 1 proposed *t*-tests on waking sAA slope and on the day-to-day change in waking sAA slope, comparing those observed before the first HLD versus those observed after the first HLD. We observed large between-participant variability in the waking sAA slope; therefore we then used a linear mixed model to investigate the hypothesized between-group comparisons while also controlling for the participant effect. The significance of the participant effect was evaluated using a likelihood ratio test to compute the fixed and random models, wherein a significant result indicates a significant participant effect.

Hypothesis 2 employed a one-factor between-participant ANOVA to test for the effect of repeated HLDs (zero vs. one vs. two or more) on two different dependent variables: (1) the waking sAA slope on the morning after the workshop ended, and (2) the pre-post change in waking sAA slope. However, only one participant completed the workshop having one HLD, and ANOVA cannot be conducted when a group contains only one observation. Thus, in order to investigate the proposed hypothesis assessing the effect of repeated HLDs, we used a linear mixed model to predict the daily waking sAA slope from the cumulative HLD count and included participant as a random factor. The variable ‘cumulative HLD count’ is a running count of the number of HLDs experienced by each participant.

Hypothesis 3 employed two Pearson’s correlation analyses to investigate the associations between HLD total intensity and, respectively, waking sAA slope and pre-post change in waking sAA slope. The variable ‘HLD total intensity’ was computed for each participant on each day, as the sum of the DLQ scores over the HLDs experienced on the previous night. This novel method of aggregating HLDs was devised with the aim of creating a summary measure that synthesizes the number of HLDs experienced and their respective intensities into a single variable that captures the overall intensity of dream-based healing.

Hypothesis 4 proposed data visualizations to explore patterns in waking sAA slope over the duration of the workshop.

## Results

Participant demographics and PTSD scores are listed in Table 1. All participants reported numerous traumatic events during their lifetime and continued use of medications and supplements throughout the workshop period (for details, see Supplementary material). Consistent with the findings of the randomized controlled study (Yount et al., 2024), this subgroup of participants experienced remarkable relief from their PTSD symptoms (see Table 1).

**Table 1:** Participant (*n* = 20) demographics and pre-post PTSD symptom scores.

Measure	Units/categories	Values - mean (SD) or n (%)
Age (19)	Years	47.1 (15.02)
Education (19)	Years	17.2 (2.8)
Sex (20)	Female	17 (85%)
	Male	3 (15%)
	Other	0 (0%)
Race (19)*	Native American	0 (0%)
	Asian	0 (0%)
	African	2 (10.5%)
	Hispanic	2 (10.5%)
	European	16 (84.2%)
Relationship (19)	In a Relationship	8 (42.1%)
	Not in a Relationship	11 (57.9%)
Overall Health (18)	Excellent	0 (0%)
	Very Good	4 (20.1%)
	Good	4 (20.1%)
	Fair	7 (36.8%)
	Poor	3 (15.8%)
Life Events Checklist (LEC) (20)		36.1 (13.2)
Pre-Workshop PCL-5 Scores (Average) (20)		50 (9.2)
Post-Workshop PCL-5 Scores (Average) (20)		23.8 (16.6)

Numbers of participants completing each measure are in parentheses.

*Participants could check more than one race.

**Figure 1:**
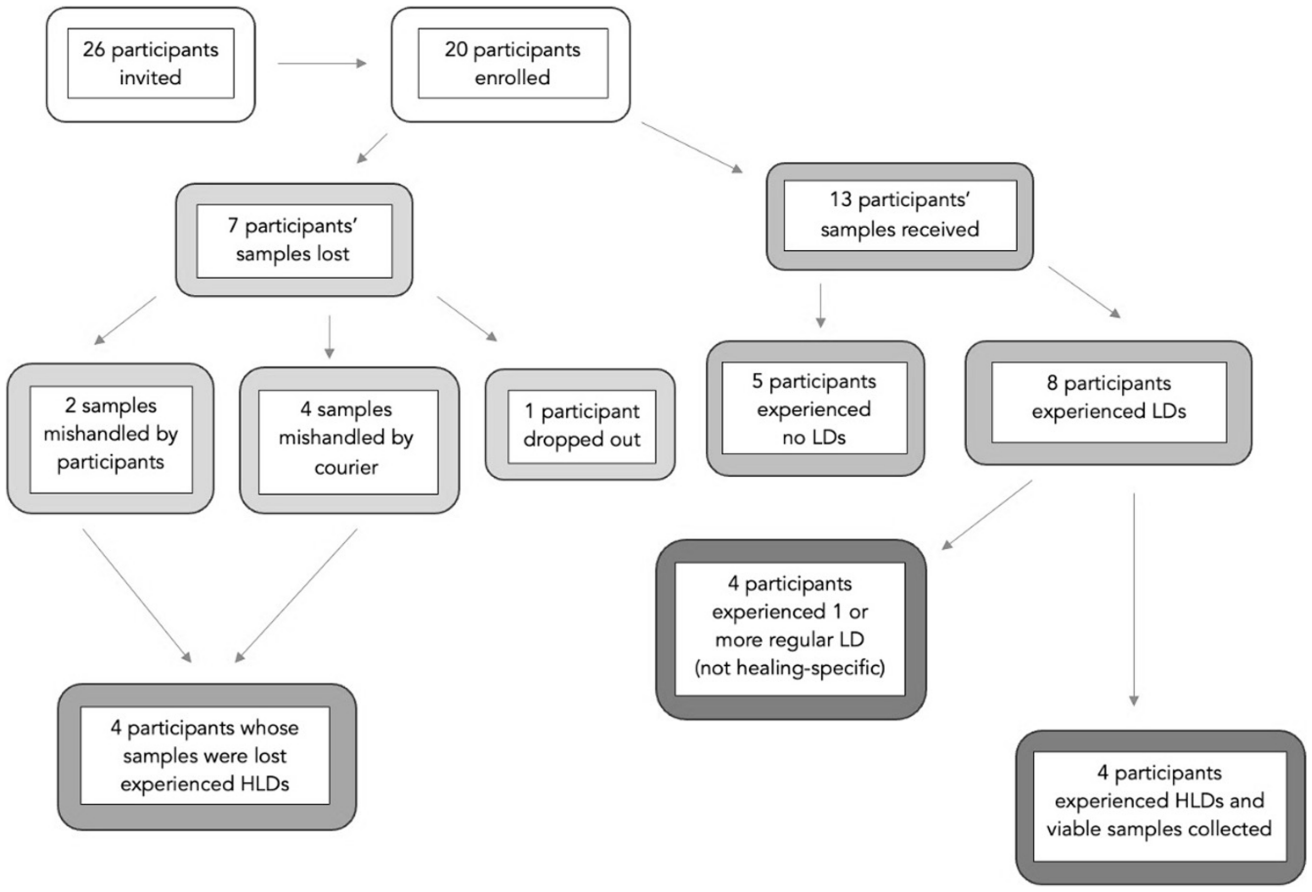
Participant samples and dreams. The term ‘samples’ refers to saliva samples participants collected on their own at home. Some samples were lost due to mishandling or participant drop-out. Participants reported each morning if they experienced no lucid dreams (no LD), a lucid dream (LD), or a healing lucid dream (HLD) during their night of sleep.

Of the 20 participants enrolled in the workshop, eight experienced a total of 18 HLDs (see Figure 1). Saliva samples were successfully obtained from 13 of the 20 participants, four of whom experienced a total of seven HLDs combined.

Substantial day-to-day variation in sAA values was evident by visual inspection of the data, including days when no LDs were reported. For instance, one participant exhibited minimal variability in waking sAA slope, irrespective of HLD occurrences. In contrast, another participant experienced consecutive LDs on days four, five, and six of the workshop, which correspond, respectively, to their highest, lowest, and second-highest waking sAA slopes observed during the week. This underscores the pervasive noise among the waking sAA slopes.

In the formal analysis of the data, we adhered to the preregistered confirmatory analysis plans for the first three hypotheses (each with two parts) and, where appropriate, adapted the analyses to suit the limited number of data points. First, an independent samples *t*-test compared all waking sAA slopes observed before the first HLD was experienced (*M* = −34.9, SE = 8.22, *n* = 74) versus all waking sAA slopes observed after the first HLD was experienced (*M* = −25.2, SE = 9.74, *n* = 21), which were not significantly different *t*(51.53) = −0.76, *p* = 0.45. After observing the large variability between participants, we employed a linear mixed model to conduct the same between-group comparisons while also including participant as a random factor to account for the dependent observations within participant. The model revealed a non-significant difference between the groups, *F*(1, 74.47) = 0.093, MSe = 3,080, *p* = 0.761. The participant effect was deemed significant by model comparison, with a likelihood ratio test statistic of 14.91, with 1 degree of freedom and value of *p* of 0.000113.

In the second part of the first hypothesis, we conducted the same analyses as in the prior paragraph except with day-to-day change in waking sAA slope as the dependent variable. The pattern of results was the same as above, except that the participant effect was nonsignificant. That is, the average day-to-day change in waking sAA slope before the first HLD (*M* = 8.08, SE = 12.42, *n* = 60) was not significantly different than after the first HLD (*M* = 3.47, SE = 12.83, *n* = 19), *t*(53.28) = 0.26, *p* = 0.80.

**Figure 2:**
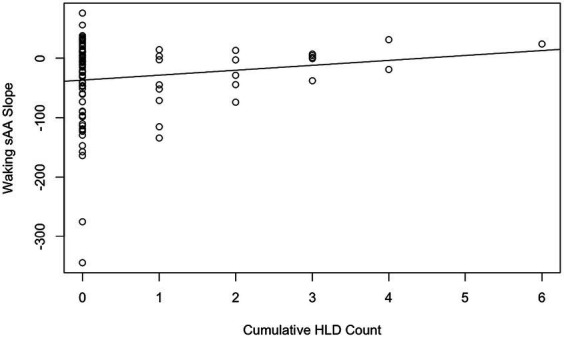
Cumulative HLD count by waking sAA slope with the line of best fit. Each data point corresponds to a daily observation (98 total, 4 removed for missing slope data).

Hypothesis 2 aimed to assess two perspectives on the effect of repeated HLDs. We first used a linear mixed model to predict for each day the waking sAA slope from the cumulative HLD count, with participant treated as a random factor. The beta value for cumulative HLD count was estimated as 5.83 (see Figure 2), which was not significantly different from zero, *t*(80.87) = 0.86, *p* = 0.39. Although the estimated slope of 5.83 was deemed nonsignificant, it reflects the trend that more HLDs were associated with higher slope values. Notably, the participant effect was significant with a likelihood ratio test statistic equal to 13.96, one degree of freedom, and corresponding *p* = 0.00019.

For the second part of Hypothesis 2, we used a linear model to predict each participant’s pre-post change in waking sAA slope from their cumulative HLD count. Results indicated a nonsignificant beta of 13.516, *t*(11) = 1.972, *p* = 0.074. A mixed model was not needed for this part because each data point here corresponds to a person, and therefore, we could not include participant as a random factor. Importantly, only four out of the 13 participants with intact saliva samples experienced HLDs, and the fact that nine participants had zero total HLDs results in this participant sample not meeting the homoscedasticity assumption, which limits interpretability.

**Figure 3:**
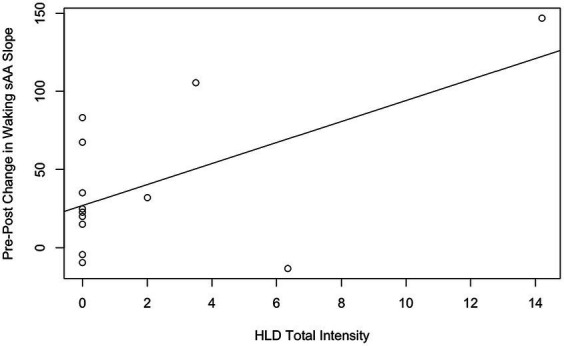
Correlations between HLD total intensity and pre-post change in waking sAA slope, with the line of best fit. The solid line is the line of best fit for all *n* = 13 participants.

Hypothesis 3 tested two correlations between the 13 participants with intact saliva samples: the correlation between HLD total intensity and waking sAA slope at the end of the workshop, and the correlation between HLD total intensity and pre-post change in waking sAA slope. The first correlation was nonsignificant, with a correlation coefficient of 0.23, *t*(11) = 0.77, *p* = 0.46. The second correlation (see Figure 3) was significant, with a correlation coefficient of 0.58, *t*(11) = 2.38, *p* = 0.036.

For Hypothesis 4, exploratory data visualizations were conducted, but none met the criteria for publication and, as a result, are not featured in this paper.

## Discussion

Alpha-amylase levels in saliva have a diurnal profile pattern that has been shown to be atypical for traumatized individuals, including those experiencing PTSD. The diurnal profile of sAA levels observed with hourly samplings from morning to evening typically shows a pronounced decrease within 1 h after awakening and a steady increase in activity during the course of the day for healthy individuals (Nater et al., 2007). Several studies have found that this sAA waking response is disrupted in traumatized populations, however. Thoma et al. (2012), for example, compared sAA diurnal profiles between a group of Bosnian War refugees with PTSD and a healthy control group and found that those with PTSD showed an awakening response profile that was opposite to those seen in healthy controls, i.e., an increase instead of a sharp decrease. Similarly, Keeshin et al. (2015) found that higher overall morning concentrations of sAA in sexually abused girls are associated with overall PTSD severity as well as symptoms of hyperarousal and intrusive symptoms, leading the authors to conclude that an increased sAA awakening response may reflect symptom-linked increases in autonomic nervous system activity.

Our rationale for this exploratory study was informed by a report suggesting that the atypical waking sAA response observed for traumatized individuals can be attenuated by mind–body interventions (Lipschitz et al., 2013), as well as by our pilot study (Yount et al., 2023). In a randomized controlled trial in which diurnal sAA profiles were measured in cancer survivors before and after experimental mind–body interventions, Lipschitz et al. (2013) found that mean sAA levels declined upon awakening post-intervention compared with survivors who did not experience an intervention. This relative decline in waking sAA levels was correlated with the efficacy of the mind–body intervention and consistent with the preliminary results of our HLD pilot study assessing sAA slope before and after a HLD workshop (Yount et al., 2023). Unexpectedly, these published results are somewhat in contrast to the weak statistical evidence found in this study, i.e., overall, a nonsignificant trend of increased waking sAA slope associated with increasing cumulative HLDs.

While we did find a significant correlation between HLD total intensity and pre-post change in waking sAA slope, we interpret this with caution. First, the scatterplot between these variables reveals a heteroscedastic distribution, as nine of the 13 participants reported zero HLDs. Moreover, the observed correlation was primarily driven by a single participant who experienced six HLDs. Taken together, we consider this significant correlation within the wider set of essentially null results. In other words, we conclude that we were unable to detect the hypothesized effect that HLDs would decrease waking sAA slope. This could be explained by many possible factors: in-home saliva collection proved problematic (discussed further below), within-participant variability was substantially larger than expected, and participants experienced fewer HLDs than anticipated. All of these factors likely played a role in reducing our power to detect the hypothesized effects of HLDs.

The high variability observed in waking sAA levels posed a challenge, primarily due to the constraints of a small sample size, which made it difficult to discern significant patterns within the data at hand. The major contributing factors leading to the reduced number of data points in the dataset were complications that arose in the context of at-home sample collection and the subsequent loss of samples due to mishandling during the shipping process. Thus, these results emphasize the value of collecting saliva samples within a controlled laboratory environment for evaluating post-awakening sAA levels. While laboratory-based experimentation was not possible for this study due to the occurrence of the COVID-19 epidemic, in-person observation would have allowed for additional improvements in the protocol, such as eye signal verification of lucidity (LaBerge et al., 1981) and real-time dialogue between experimenters and dreamers during rapid eye movement sleep (Konkoly et al., 2021). Additionally, the participants were given the freedom to use any technique that they were introduced to as they wished, which resulted in an organic heterogeneity to the actual induction techniques that each participant followed. Likewise, the integration of lucid dreaming training with the elements of group psychotherapy prevents conclusions from being drawn that are specific to dreaming elements alone. Further, the study did not control for medication and supplement use among the participants throughout the workshop. Some of these taken at specific times may have affected the sAA measurements and other outcomes.

In conclusion, this study endeavored to identify a biomarker associated with the therapeutic potential of HLDs by measuring waking sAA levels in participants experiencing relief from PTSD symptoms. High-quality saliva samples were collected from only four participants who also reported achieving dream lucidity and remembering their intention to invoke healing in the dream, but analysis of waking sAA slopes revealed that day-to-day variability in waking sAA slopes – even on non-lucid dream days – made it challenging to detect any significant impact of HLDs. The small sample size was partially due to the elusiveness of dream lucidity but also contributed to by mishandling of samples during transport and other complications related to at-home sample collection. Thus, the results did not provide support for the hypothesis that HLDs lead to consistent changes in the waking sAA slope in individuals with PTSD symptoms and underscore the importance of conducting saliva sample collection in a controlled laboratory environment for assessing sAA awakening responses.

## Data availability statement

The datasets presented in this study can be found in online repositories. The names of the repository/repositories and accession number(s) can be found in the article/Supplementary material.

## Ethics statement

The studies involving humans were approved by the Institute of Noetic Sciences Institutional Review Board. The studies were conducted in accordance with the local legislation and institutional requirements. The participants provided their written informed consent to participate in this study.

## Author contributions

GY: Conceptualization, Funding acquisition, Investigation, Methodology, Project administration, Writing – original draft, Writing – review & editing. ST: Conceptualization, Investigation, Writing – review & editing. TS: Conceptualization, Methodology, Writing – review & editing. MK: Conceptualization, Data curation, Formal analysis, Methodology, Writing – review & editing. HW: Conceptualization, Methodology, Writing – review & editing.
