# Supplementary material for: Trends in waking salivary alpha-amylase levels following healing lucid dreams
Source: Front Psychol. 2024 Jan 17;15:1347499. doi: 10.3389/fpsyg.2024.1347499 (PMC10828844; doi:10.3389/fpsyg.2024.1347499)
Supplement: Supplementary file 1 [file Data_Sheet_1.docx]

**Supplementary Material**

Lucid Dream Definition and Example

Lucid dreaming is a special sort of dream in which you know that you are dreaming while still in the dream. Typically, you tell yourself “I’m dreaming!” or “This is a dream!” This realization often (but not necessarily) leads to the ability to deliberately control one’s actions or to observe passively the course of the dream with full conscious awareness, similar to the awareness you have while awake.

An example of a lucid dream is as follows: “It was snowing gently. I was alone on the rooftop of the world, climbing K2. As I made my way upward through the steeply drifting snow, I was astonished to notice my arms were bare: I was wearing a short-sleeved shirt, hardly proper dress for climbing the second-highest mountain in the world! I realized at once that the explanation was that I was dreaming! I was so delighted that I jumped off the mountain and began to fly away …”

Trauma Exposure

Regarding the following events, the percentages of participants that had exposure to a category of events were: 67% for natural disaster (e.g., earthquake), 56% for fire or explosion, 78% for transportation accident (e.g., car accident), 67% for serious accident, 22% for exposure to toxic substance, 89% for physical assault (e.g., beaten up), 44% for assault with a weapon, 78% for sexual assault, 78% for other unwanted or uncomfortable sexual experiences, 56% combat or exposure to a war-zone experience, 44% for captivity (e.g., prisoner of war), 78% for life-threatening illness or injury, 67% for severe human suffering, 78% for sudden violent death, 67% sudden accidental death, 33% for serious injury, harm, or death you caused to someone else, and 67% for any other very stressful event or experience. Scores listed above reflect either direct experience, witnessing, or hearing about each event to a degree that was traumatic to the individual.

Medication and Supplement Usage

The participants were not asked to alter their typical use of medications and supplements during the workshop. The following is a list of the self-reported medications and supplements used, with the number of participants reporting use indicated within parentheses: 5HTP (1), Antacids (2), Aprolazam (1), Armour Thyroid (3), BCAA's (1), Berberine (1), Bilberry extract (1), Blueberry Juice (1), Buspirone (1), Calcium (1), Clonazepam (1), Creatine (1), Liquid Minerals (1), Entresto (1), Escitalopram (1), Estradiol (1), Gabapentin (1), Hawthorne (1), Herbal Adrenal Supplement (1), Hydrochlorothiazide (1), Hydroxyzine (1), Ibuprofen (1), Levothyroxine (1), Lisinopril (1), Magnesium (3), Melatonin (1), Methadone (1), Metoprolol (1), Multivitamin (2),

Niacinimide (1), Ocrevus (1), Omega Fish oil (1), Papaya Enzymes (1), Promethazine (1), Propranolol (2), Psilocybin microdose (1), Superfood Mushroom powder (2), Trazadone (2), Valarian (1), Valium (2), Venflaxine (1), Vitamin C (2), Vitamin D (2), Zinc (1). Many of the participants reported using more than one medication or supplement, and seven (7) reported using none.

Additional References (Supplementary Materials)

Aron, A., Aron, E. N., & Smollan, D. (1992). Inclusion of other in the self scale and the structure of interpersonal closeness. Journal of Personality and Social Psychology, 63(4), 596.

Cappelleri, J. C., Bushmakin, A. G., McDermott, A. M., Sadosky, A. B., Petrie, C. D., & Martin, S. (2009). Psychometric properties of a single-item scale to assess sleep quality among individuals with fibromyalgia. Health and Quality of Life Outcomes, 7(1), 54. https://doi.org/10.1186/1477-7525-7-54

Cloninger, C. R., Przybeck, T. R., Svrakic, D. M., & Wetzel, R. D. (1994). The Temperament and Character Inventory (TCI): A guide to its development and use. Center for Psychobiology of Personality, Washington University.

Kelly, J., 2002. The Way of the Dreamcatcher: Spirit Lessons with Robert Lax: Poet, Peacemaker, Sage. The Acorn, 11(2), pp.43-44.

Nielsen, T., & Powell, R. A. (2015). Dreams of the Rarebit Fiend: Food and diet as instigators of bizarre and disturbing dreams. Frontiers in Psychology, 6, 47. https://doi.org/10.3389/fpsyg.2015.00047

Schultz, P. W., Shriver, C., Tabanico, J. J., & Khazian, A. M. (2004). Implicit connections with nature. Journal of Environmental Psychology, 24(1), 31–42. https://doi.org/10.1016/S0272-4944(03)00022-7
